# The immunogenicity to the first anti-TNF therapy determines the outcome of switching to a second anti-TNF therapy in spondyloarthritis patients

**DOI:** 10.1186/ar4258

**Published:** 2013-07-26

**Authors:** Chamaida Plasencia, Dora Pascual-Salcedo, Sara García-Carazo, Leticia Lojo, Laura Nuño, Alejandro Villalba, Diana Peiteado, Florencia Arribas, Jesus Díez, Maria Teresa López-Casla, Emilio Martín-Mola, Alejandro Balsa

**Affiliations:** 1 Rheumatology Department, La Paz University Hospital-Idipaz, Paseo la Castellana 261, Madrid, PC 28046, Spain; 2 Immunology Unit, La Paz University Hospital-Idipaz, Paseo la Castellana 261, Madrid, PC 28046, Spain; 3 Statistic Department, La Paz University Hospital-Idipaz, Paseo la Castellana 261, Madrid, PC 28046, Spain

## Abstract

**Introduction:**

Anti-TNF drugs have proven to be effective against spondyloarthritis (SpA), although 30% of patients fail to respond or experience adverse events leading to treatment discontinuation. In rheumatoid arthritis, the presence of anti-drug antibodies (ADA) against the first TNF inhibitor influences the outcome after switching. Our aim was to assess whether the response to a second anti-TNF drug is related to the previous development of ADA to the first anti-TNF drug SpA patients.

**Methods:**

Forty-two SpA patients began a second anti-TNF drug after failing to respond to the first anti-TNF therapy. Clinical activity was assessed by the Ankylosing Spondylitis Disease Activity Score (ASDAS) at baseline (at the beginning of the first and second anti-TNF therapy) and at 6 months after switching. The drug and ADA levels were measured by ELISA before each administration.

**Results:**

All patients were treated with anti-TNF drugs and mainly due to inefficacy were switched to a second anti-TNF drug. Eleven of 42 (26.2%) developed ADA during the first biologic treatment. At baseline, no differences in ASDAS were found in patients with or without ADA to the first anti-TNF drug (3.52 ± 1.03 without ADA vs. 3.14 ± 0.95 with ADA, *p *= 0.399) and to the second anti-TNF drug (3.36 ± 0.94 without ADA vs. 3.09 ± 0.91 with ADA, *p *= 0.466). At 6 months after switching, patients with previous ADA had lower disease activity (1.62 ± 0.93 with ADA vs. 2.79 ± 1.01 without ADA, *p *= 0.002) and most patients without ADA had high disease activity state by the ASDAS (25 out of 31 (80.6%) without ADA vs. 3 out of 11 (27.3%) with ADA, *p *= 0.002).

**Conclusions:**

In SpA the failure to respond to the first anti-TNF drug due to the presence of ADA predicts a better clinical response to a second anti-TNF drug.

## Introduction

Spondyloarthritis (SpA) describes a group of diseases including ankylosing spondylitis (AS), psoriatic SpA, SpA related to inflammatory bowel disease (IBD), reactive arthritis, a subgroup of juvenile idiopathic arthritis and nonradiographic axial spondyloarthritis [1]. Several studies have demonstrated the efficacy of biological agents, such as anti-TNFα drugs, for treating SpA patients [2-9].

The available anti-TNF drugs differ in chemical structure, half-life, route of application and capacity to induce immunogenicity, and they also have somewhat different mechanisms of action [10,11]. Although the efficacy of anti-TNF drugs against SpA has been shown in large, randomised clinical trials [6,12-16], it is known that some patients fail to respond to treatment or experience adverse events necessitating treatment discontinuation [11,17]. Part of this treatment failure can be explained by the development of anti-drug antibodies (ADA) [17-20].

To date, only two studies have been published that correlate the clinical response and immunogenicity to anti-TNF drugs in rheumatoid arthritis (RA) patients who switched to a second anti-TNF drug [21,22]. In these studies, RA patients with ADA against the first anti-TNF drug have been shown to have a better clinical response after switching to a second anti-TNF therapy than patients who did not develop ADA against the first anti-TNF drug [21,22]. Until now, no data have been published about the association between immunogenicity to the first anti-TNF drug and the clinical response after switching to a second anti-TNF drug in SpA patients. In this study, we analysed whether the clinical response to a second anti-TNF drug is conditioned by the development of ADA against the first anti-TNF drug in a group of SpA patients.

## Materials and methods

### Patients and sera

A total of 42 SpA patients (27 AS, 10 nonradiographic axial SpA, 2 SpA associated with IBD, 2 psoriatic SpA and 1 SpA secondary to reactive arthritis) without previous biological treatment were included. All of these patients had axial involvement and most of them had some peripheral articular manifestation as dactylitis, enthesopathy, monoarthritis and oligoarthritis (28/42 (66.7%) SpA patients: 13 AS, 10 nonradiographic axial SpA, 2 psoriatic SpA, 2 SpA related to IBD and 1 reactive arthritis. The patients were enrolled at the Department of Rheumatology of La Paz University Hospital. This was an ambispective observational study that was approved by the La Paz Hospital Ethics Committee, and all patients provided informed written consent. The retrospective study period covered the years 2005 to 2008, and the prospective study period covered 2009 to 2011. All of the AS patients fulfilled the New York revised criteria for AS [23]. The psoriatic arthritis patients fulfilled the GRAPPA group criteria [24].

All patients received anti-TNF drugs as a first biological treatment (infliximab (Ifx), adalimumab (Ada) and etanercept (Etn)) and later switched to a second anti-TNF drug (Ifx, Ada, Etn and golimumab (Gol)). The selection of all anti-TNF drugs was left to the discretion of the physician, with consideration of patient characteristics, type of disease, and patient preference. Owing to the observational design of the study, no specific criteria for drug withdrawal were required, and the diagnoses of treatment failure and adverse events were based on the judgement of the treating physician. Ifx was administered intravenously at 5 mg/kg at 0, 2 and 6 weeks and every 8 weeks thereafter, and the remaining anti-TNF drugs were administrated subcutaneously (Ada, 40 mg/2 weeks; Etn, 50 mg/week; and Gol, 50 mg/month).

Disease activity was measured by the Ankylosing Spondylitis Disease Activity Score (ASDAS) [25,26] and was assessed at baseline and every 6 months. At the time of inclusion, all patients had evidence of active spinal disease, as indicated by a mean ASDAS of 3.42 ± 1.01. Clinically important improvement was defined as change in ASDAS ≥1.1 [26]. Data related to the clinical activity in the retrospective period were obtained from our database of patients on biological therapy.

Blood samples were collected a maximum of 24 hours before biological drug administration for subcutaneous anti-TNF or just before intravenous infusion for Ifx. Precise timing was required to compare the results because the drug levels in the serum can become undetectable over longer time intervals as a result of normal drug pharmacokinetics rather than the formation of immunocomplexes with ADA. All sera, including those of the retrospective period, were stored at -20°C until the drug and ADA concentrations were measured.

### Measurement of drug and anti-drug antibody concentrations

The serum drug concentrations (Ifx, Ada and Etn) were determined by sandwich ELISA, as described previously [27-29]. Serum drug levels were considered positive for Ifx if >10 ng/ml, for Ada if >5 ng/ml and for Etn if >30 ng/ml.

Serum ADA levels (antibodies to Ifx, antibodies to Ada and antibodies to Etn) were detected using a two-site (bridging) ELISA, as previously described [27-29]. The cutoff value for the presence of antibodies to Ifx was established at 50 AU/ml, for antibodies to Ada at 10 AU/ml and for antibodies to Etn at 50 AU/ml.

To determine the cutoff value of each assay, sera from 150 healthy controls and from 100 RA patients without anti-TNF treatment (70% positive for rheumatoid factor) were studied. The mean ± 6 standard deviations was used to establish cutoff points.

### Statistical analysis

The statistical analyses were performed using the Statistical Package for the Social Sciences, version 11.0 (SPSS, Chicago, IL, USA). Descriptive statistics included the mean and standard deviation or the median and interquartile range. Differences in baseline characteristics were assessed using Pearson's chi-square test and Fisher's exact test for ordinal variables and using the Mann-Whitney U test for continuous variables. The continuous data were compared between groups using the Mann-Whitney U test. Statistical significance was calculated using the log-rank test, and p < 0.05 was considered significant.

## Results

### Patient characteristics

A total of 42 SpA patients were enrolled in this study with a mean ± standard deviation age of 49.6 ± 10.4 years at the time of inclusion, and 23 (54.8%) were men. The baseline demographic and clinical characteristics of the global patient population, categorised according to future ADA development against the first anti-TNF therapy, are shown in Table 1. No differences in patient characteristics were present at baseline between those who later developed ADA and those who did not (Table 1).

**Table 1:** Demographic characteristics of 42 spondyloarthritis patients

Characteristic	Total (42 patients)	Without ADA (31 patients)	With ADA (11 patients)	*p *value
Sex, male	23 (54.8%)	16 (51.6%)	7 (63.6%)	0.726
Age	49.60 ± 10.46	51.26 ± 10.08	44.91 ± 10.53	0.084
HLA-B27-positive^a^	23/36 (64%)	17/27 (63%)	6/9 (66.7%)	0.841
Disease duration (years)	12.24 ± 8.23	11.61 ± 8.04	14 ± 8.92	0.416
Baseline ASDAS	3.42 ± 1.01	3.52 ± 1.03	3.14 ± 0.95	0.399
Concomitant treatment				
Methotrexate	9 (21.5%)	8 (25.8%)	1 (9.1%)	0.498
Other DMARDs	10 (23.8%)	5 (16.1%)	5 (45.4%)	0.115
Methotrexate + other	1 (4,7%)	2 (6.4%)	0 (0%)	0.599
DMARDs				
Monotherapy	21 (50%)	16 (51.7%)	5 (45.5%)	0.126
Corticosteroid therapy	15 (35.7%)	9 (29%)	6 (50%)	

Data presented as *n *(%) or mean ± standard deviation. ADA, anti-drug antibodies; ASDAS, Ankylosing Spondylitis Disease Activity Score; DMARD, disease-modifying anti-rheumatic drug. ^a^*n*/total number (%).

### Immunogenicity in relation to biological therapy

All 42 patients were treated with an anti-TNF drug as the first biologic therapy (20 with Ifx, 5 with Ada and 17 with Etn) and were switched to a second anti-TNF treatment (9 Ifx, 19 Ada, 8 Etn and 6 Gol) due to inefficacy (39 out of 42 patients, 92.8%; 11 of them ADA-positive) and/or adverse events (8 out of 42 patients, 19%). Of the eight patients who withdrew due to adverse events, three had been treated with Ifx (all of them with ADA and clinical inefficacy, having infusion-related reactions) and five with Etn (two out of five patients also had clinical inefficacy, all of them having local injection reaction and/or pruritus).

ADA were detected in 11 (26.2%) patients (7/27 (25.9%) AS, 3/10 (30%) undifferentiated SpA and 1/2 (50%) SpA related to IBD) during treatment with the first anti-TNF drug and were more frequent in patients treated with Ifx (9 (81.8%) with Ifx, 2 (18.2%) with Ada, 0 (0%) with Etn, *p *= 0.006). ADA appeared mainly within the first year of anti-TNF therapy (mean ± standard deviation: 12.89 ± 5.92 months), except for five patients in whom ADA were detected at 18 months (2 patients), 20 months (2 patients) and 28 months (1 patient). At 6 months after switching to the second anti-TNF drug, ADA were detected in only two patients treated with Ifx, who had not previously developed antibodies against the first anti-TNF drug. The drug and ADA concentrations were not evaluated for Gol.

Most patients without ADA (28/31) against the first anti-TNF drug had clinical inefficacy and detectable serum drug levels just before the switch to the second anti-TNF drug (median (interquartile range): 3,008 (680 to 3,076) ng/ml for Ifx, 3,072 (2,048 to 4,096) ng/ml for Ada, 1,111 (683 to 2,077) ng/ml for Etn), so these patients were considered as having a primary inefficacy. Only three patients treated with Etn without detectable antibodies before switching did not have loss of efficacy and they were switched due only to adverse events. Furthermore, all of 11 patients with ADA against the first anti-TNF treatment had loss of efficacy and undetectable drug levels before switching, so they were classified as having secondary inefficacy related to development of immunogenicity.

### Clinical response in relation to immunogenicity

At baseline, no differences in disease activity were observed between patients who did or did not later develop ADA against the first anti-TNF drug (baseline ASDAS first anti-TNF: 3.52 ± 1.03 without ADA vs. 3.14 ± 0.95 with ADA, *p *= 0.399; baseline ASDAS second anti-TNF: 3.36 ± 0.94 without ADA vs. 3.09 ± 0.91 with ADA, *p *= 0.466). Also, there were no differences in clinical activity at baseline to the first and second anti-TNF drugs in patients with and without ADA (without ADA: 3.52 ± 1.03 to the first anti-TNF vs. 3.36 ± 0.94 to the second anti-TNF, *p *= 0.383; with ADA: 3.14 ± 0.95 to the first anti-TNF vs. 3.09 ± 0.91 to the second anti-TNF, *p *= 0.922).

**Figure 1:**
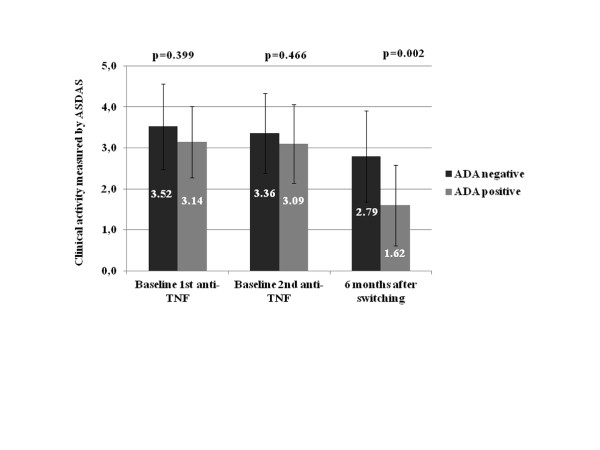
**Association between clinical activity (Ankylosing Spondylitis Disease Activity Score) and immunogenicity**. Ankylosing Spondylitis Disease Activity Score (ASDAS; mean ± standard deviation) measured at baseline (first and second anti-TNF drugs) and at 6 months after switching to a second anti-TNF drug in patients who did or did not develop anti-drug antibodies (ADA) against the first anti-TNF drug.

**Figure 2:**
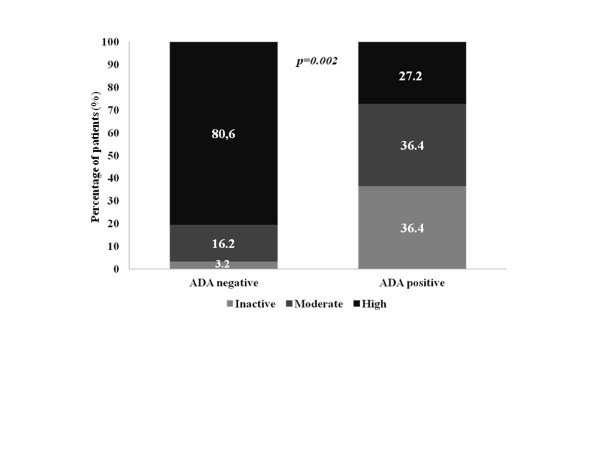
**Association between anti-drug antibody status and clinical activity (Ankylosing Spondylitis Disease Activity Score) after switching**. Clinical activity classified into inactive (light shading), moderate (medium shading) and high/very high activity (dark shading), according to Ankylosing Spondylitis Disease Activity Score criteria, 6 months after switching to a second anti-TNF drug in patients who had developed anti-drug antibodies (ADA) against the first anti-TNF drug.

At 6 months after switching, the patients who had developed ADA against the first anti-TNF drug had lower disease activity, as measured by the ASDAS (1.62 ± 0.93 with ADA vs. 2.79 ± 1.01 without ADA, *p *= 0.002) (Figure 1), and more patients had inactive disease (4 out of 11 (36.4%) with ADA vs. 1 out of 31 (3.2%) without ADA, *p *= 0.002) (Figure 2). After 6 months of switching, most patients without ADA against the first anti-TNF drug were classified as being in a high or very high disease activity state by the ASDAS (25 out of 31 (80.6%) without ADA vs. 3 out of 11 (27.3%) with ADA, *p *= 0.002) (Figure 2).

**Figure 3:**
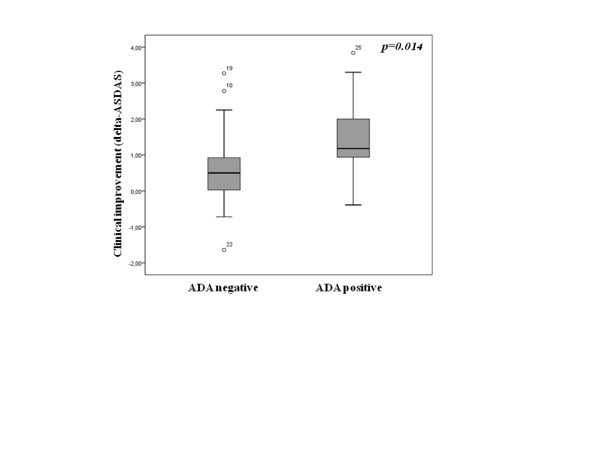
**Association between clinical improvement (change in Ankylosing Spondylitis Disease Activity Score) and immunogenicity**. Change in Ankylosing Spondylitis Disease Activity Score (delta-ASDAS) measured 6 months after switching in spondyloarthritis patients who presented or not anti-drug antibodies (ADA) against the first anti-TNF. Data shown as interquartile ranges (p75, upper edge; p25, lower edge; p50, midline of the box).

At 6 months after switching we observed a greater clinical improvement, as measured by change in ASDAS, in patients with ADA as compared with those without ADA (1.49 ± 1.27 with ADA vs. 0.56 ± 1.01 without ADA, *p *= 0.014) (Figure 3). A total of 13 patients achieved clinically relevant improvement, and clinical improvement was more frequent in patients who had developed ADA (8t out of 11 (72.7%) with ADA vs. 5 out of 31 (16.1%) without ADA, *p *= 0.001). Three out of the five patients without ADA who had an important clinical improvement after switching were treated with Etn as the first anti-TNF drug and the reason for change to a second anti-TNF drug was adverse effects.

When a subanalysis is performed taking only the group of patients with AS (*n *= 27), we observed that our results in relation to clinical activity and immunogenicity are consistent with those observed analysing all 42 patients. At baseline of the first and second anti-TNF drugs, no differences were seen in clinical activity (ASDAS) between patients with and without ADA (baseline first anti-TNF: 3.61 ± 1.11 without ADA vs. 3.57 ± 0.83 with ADA, *p *= 0.929; baseline second anti-TNF: 3.43 ± 0.77 without ADA vs. 2.87 ± 1.01 with ADA, *p *= 0.136). However, 6 months after switching the clinical activity was lower in patients with previous ADA (2.78 ± 1.05 without ADA vs. 1.38 ± 0.75 with ADA, *p *= 0.0.004).

No differences were observed in clinical activity and clinical improvement between patients treated with a second anti-TNF drug with a mAb or fusion protein (ASDAS after 6 months of switching: 2.47 ± 1.12 with mAb vs. 2.53 ± 1.11 with fusion protein, *p *= 0.908; change in ASDAS after 6 months of switching: 0.73 ± 1.17 with mAb vs. 1.13 ± 1.03 with fusion protein, *p *= 0.372).

## Discussion

In this article we studied the role of immunity against a first anti-TNF drug in the short-term response to a second anti-TNF drug in a group of SpA patients. We show that patients in whom drug discontinuation was associated with ADA development achieved a better clinical response at 6 months after switching than patients who had not developed ADA.

Anti-TNF drugs are the only biological therapies available to treat SpA patients with an inadequate response to conventional treatment, and their efficacy has been demonstrated in several randomised placebo-controlled studies [2-9]. However, a number of patients fail to respond or experience adverse events necessitating treatment discontinuation [11,17,20,30,31]. Thus, it is crucial to know what factors predict the treatment response in SpA patients. Different predictors of a favourable response to the first anti-TNF drug have been reported in the literature, including shorter disease duration, younger age, HLA-B27 positivity, a lower Bath Ankylosing Spondylitis Functional Index score, higher C-reactive protein levels, a higher Bath Ankylosing Disease Activity Index score, male sex and the presence of peripheral arthritis and spinal inflammation on magnetic resonance imaging [18,32-39]. These data were not analysed in the present study because we only recruited SpA patients who had discontinued their first anti-TNF drug.

Only a few prospective studies have reported detailed information about switching to a second anti-TNF drug in AS patients [11,40,41]. One recent publication showed that switching to a second anti-TNF drug could benefit some AS patients, and up to one-third of patients achieved a good response [11]. However, disease activity at 3 months after switching was generally worse for switchers on their second anti-TNF drug than for nonswitchers [11]. Similar findings were observed in another study that included 1,250 AS patients, 326 of whom had previously received an anti-TNF drug [37]. In this study, anti-TNF-naïve AS patients achieved greater treatment responses than patients who switched to a second anti-TNF drug [37].

The immunogenicity of biological therapies has been shown to influence secondary inefficacy in rheumatic diseases [17,28,42-52]. The frequency of ADA development in SpA patients varies between different studies (25.5 to 29% for antibodies to Ifx and 31% for antibodies to Ada) [17,19,20,49-51]. Several publications have described the relationship between the development of ADA and the clinical response in SpA patients [17,19,20,52-54]. In previous work conducted by our group, antibodies to Ifx were detected in 25.5% of SpA patients treated with Ifx, and a strong correlation was observed between antibodies to Ifx development and clinical response as measured by the ASDAS [49]. de Vries and colleagues observed that 31% of AS patients treated with Ada developed antibodies to Ada, and most of the patients did not reach an ASAS response after 6 months of treatment [20]. In this study, 26.2% (11/42) of patients who discontinued the first anti-TNF drug had detectable ADA, and most had exhibited a good response to therapy until ADA development. However, the majority of SpA patients without ADA who switched to a new anti-TNF drug never demonstrated clinical improvement, and indeed they typically had a detectable serum drug concentration. These findings may suggest that TNF is not the main cytokine instigating disease activity in these patients or that symptoms in these patients are not related to the inflammatory activity of the disease.

Currently, there are only two reports that relate immunogenicity status to the first anti-TNF drug and its clinical response after switching to a second anti-TNF drug in RA patients [21,22]. Bartelds and colleagues observed that RA patients who had developed antibodies to Ifx against the first anti-TNF drug had no significant differences in clinical improvement after switching (change in Disease Activity Score for 28 joints) when compared with anti-TNF-naïve patients [21]. Nevertheless, switchers without antibodies to Ifx exhibited a significantly lower clinical response than naïve RA patients [21]. Similar findings were reported in a subsequently published study in RA patients (naïve and switchers) treated with Etn [22], which demonstrated that naïve patients and switchers with ADA had a greater clinical response than did patients who switched without ADA [22]. To our knowledge, the present work is the first report in which the influence of immunogenicity against the first anti-TNF drug has been associated with clinical activity after switching in SpA patients. As shown above, clinical improvement was greater in patients who switched after developing ADA, and 73% of patients who achieved clinical improvement after switching had developed ADA to the previous anti-TNF drug.

Our study has several limitations. The number of patients is relatively small because all patients came from the same centre, and SpA patients are less likely than RA patients to discontinue anti-TNF drug [38,41]. However, our results show differences in treatment outcomes that are similar to those described after switching anti-TNF drugs in RA [21,22]. Furthermore, the decision to stop therapy or to change drugs was not standardised because it was based on the decision of the responsible rheumatologist. This may explain why disease activity, although not significantly different at the baseline of the second anti-TNF drug, was slightly lower in the group that developed ADA, because patients are more sensitive to deterioration once they have previously improved, and the switch is performed at lower levels of disease activity. However, it is important to highlight that this is the pattern normally used to determine whether a therapeutic change is required in clinical practise. Finally, different anti-TNF drugs were used in the study, both as the first and the second anti-TNF therapies, which may have influenced the results. However, no differences in the effectiveness of the three most commonly used anti-TNFs have been demonstrated in clinical practice in AS [55], so it is unlikely that this would have affected the results.

## Conclusions

Similar to RA, the failure to respond to a first anti-TNF drug due to the development of ADA predicts a better clinical response to a second biological treatment in SpA. The presence of ADA against the first anti-TNF drug is a determining factor for the response to a second anti-TNF drug. The study of the immunogenicity in biological treatment failure may help predict the response to a second biological treatment in SpA.

## Abbreviations

Ada: adalimumab; AS: ankylosing spondylitis; ASDAS: Ankylosing Spondylitis Disease Activity Score; ADA: anti-drug antibodies; ELISA: enzyme-linked immunosorbent assay; Etn: etanercept; Gol: golimumab; IBD: inflammatory bowel disease; Ifx: infliximab; mAb: monoclonal antibody RA: rheumatoid arthritis; SpA: spondyloarthritis; TNF: tumour necrosis factor.

## Competing interests

AB has received fees from Roche, Schering-Plough, Wyeth, Abbott, BMS and UCB. EM-M is a consultant and a member of speakers' bureaus for Pfizer, MSD, UCB and Abbott. ChP, DP-S and LN have received speaker honoraria from Pfizer. All other authors declare that they have no competing interests.

## Authors' contributions

ChP, DP-S, EM-M and AB wrote the article. DP-S, SGC, MTL-C and ChP carried out the data collection and databases. AV, DP, LN, LL, SGC, AB, EM-M and ChP performed the clinical evaluation of patients. JD, SGC and ChP performed the statistical analysis. DP-S and FA performed the laboratory assays. All authors read and approved the final manuscript.
